# Meibomian Gland Dysfunction: Endocrine Aspects

**DOI:** 10.5402/2011/465198

**Published:** 2011-10-20

**Authors:** Ozlem G. Sahin, Elçin Kartal, Nusret Taheri

**Affiliations:** ^1^Department of Ophthalmology, College of Health Sciences, Middle East Technical University, 06800 Ankara, Turkey; ^2^Department of Statistics, Middle East Technical University, 06800 Ankara, Turkey; ^3^Department of Biochemistry, College of Health Sciences, Middle East Technical University, 06800 Ankara, Turkey

## Abstract

*Purpose*. To compare the hormone levels of patients with seborrheic meibomian gland dysfunction with controls. *Procedures*. This is a retrospective case-control study involving 50 patients and 50 controls. Blood workup for hormones was studied in both groups by using macroELISA (enzyme-linked immunosorbent assay). Statistical evaluation was done by using SPSS 15.0 independent samples *t*-test. *Results*. There were statistically significant differences of serum testosterone and dehydroepiandrosterone sulphate levels between patients and controls (*P* = 0.000). Female gender showed statistically significant differences of serum thyroid-stimulating hormone and prolactin levels between patients and controls (*P* = 0.014 and *P* = 0.043), in addition to serum testosterone and dehydroepiandrosterone sulphate levels (*P* = 0.000 and *P* = 0.001). However, male gender showed statistically significant differences of only serum testosterone and dehydroepiandrosterone sulphate levels between patients and controls. (*P* = 0.003 and *P* = 0.003 resp.). *Conclusions*. Increased serum levels of testosterone and dehydroepiandrosterone sulphate in both genders should be considered as diagnostic markers for seborrheic meibomian gland dysfunction.

## 1. Introduction

Meibomian gland dysfunction (MGD) is considered to be a discrete disease entity without prominent inflammatory alterations of the lid margins and a frequent cause of wetting deficiencies of the ocular surface leading to dry eye disease [1]. MGD is grouped as obstructive and seborrheic dysfunction [2, 3]. Obstructive MGD is characterized by hyperkeratinization of the ductal epithelium and increased viscosity of the meibum resulting in obstruction of the meibomian gland duct and orifice [1, 2]. Obstructive MGD is reported to be much more frequent in the general population and increases with age [2]. Seborrheic MGD is characterized by hypersecretion of meibum [3]. Proposed diagnostic criteria for seborrheic MGD include ocular symptoms and lid margin abnormalities [3]. Age, hormonal disturbances, and environmental influences have been considered in the pathogenesis of both obstructive and seborrheic MGD [2]. The effect of androgens on meibomian gland function has been studied in a group of patients with an average of 70.9 years, and androgen deficiency is considered as a critical factor in the pathogenesis of MGD and dry eye [4, 5]. Very little information exists concerning the correlation of serum levels of sex hormones, thyroid hormones, thyroglobulin, cortisol, and prolactin with seborrheic MGD between 20–30 years of age. The purpose of this study is to compare the serum levels of dehydroepiandrosterone sulphate (DHEA-S), testosterone, estrodiol, 17-hydroxyprogesterone (17-OH-Prog), prolactin, follicle-stimulating hormone (FSH), luteinizing hormone (LH), thyroid-stimulating hormone (TSH), bound and unbound thyroid hormones (T3, T4), thyroglobulin, and cortisol between patients with seborrheic MGD and controls in a gender-based design between 20–30 years of age.

## 2. Methods

This is a retrospective case-control study involving 50 patients, 31 male and 19 female with a mean ± standard deviation (SD) of 23.92 ± 5.73 years, and 50 controls 19 male and 31 female with a mean age of 23.36 ± 4.61 years. The study is approved by the Human Studies Committee of the Middle East Technical University (Ankara, Turkey) and were conducted in accordance with guidelines established by the Declaration of Helsinki. Ocular symptoms including ocular fatigue, discharge, foreign body sensation, dryness, discomfort, sticky sensation, pain, epiphoria, itching, redness, heavy sensation, glare, excessive blinking, and history of chalazion or hordeolum were scored from 0 to 14 according to the number of symptoms present [3]. Lid margin abnormalities including irregular lid margin, vascular engorgement, plugged meibomian gland orifices, and anterior or posterior replacement of mucocutaneous junction, were scored from 0 to 4 depending on the number of abnormalities present [3]. Superficial punctate keratopathy (SPK) in the cornea was scored from 0 to 3 [3, 6]. The tear film break-up time (BUT) was measured consecutively three times after instillation of fluorescein 1% (fluorescite injection) and the median value was adopted [6]. Assessment of meibomian gland function was done by using two techniques: observation of meibomian gland orifices by using biomicroscopy and transillumination observation techniques by using a light probe (meibography) [7, 8]. Meibography was performed by using a transillumination device for vitrectomy with a 20-gauge fiberoptic light probe (Bausch and Lomb Millennium, Rochester, NY) including tungsten-halogen ad metal halide. Meiboscore for upper and lower eyelids was used as previously described: grade 0 (no loss of meibomian glands), grade 1 (loss of less than one-third of the total area of the meibomian glands), grade 2 (loss of between one-third and two-thirds of the total area), and grade 3 (loss of over two-thirds of the total area) [7, 8]. Meibomian gland expression was evaluated by applying moderate digital pressure on the tarsus of the upper eyelid, and the degree of ease with which meibum secretion was induced was evaluated as grade 0 (clear meibum, easily expressed), grade 1 (cloudy meibum, expressed with mild pressure), grade 2 (cloudy meibum expressed with more than moderate pressure), and grade 3 (no meibum expressed even with hard pressure) [9]. The procedure was performed by the same ophthalmologist. Tear film production was evaluated using the Schirmer test without application of topical anesthetics. A diagnosis of seborrheic MGD was made on the basis of an ocular symptom score of 3 or more and a lid margin score of 2 or more [3, 6]. Patients with meiboscore 1 or more, a Schirmer value of 5 mm or less, the tear break-up time of less than 5 seconds, and SPK score of 1 or more, were excluded from the study. The controls had no evidence of blepharitis, seborrheic MGD, or corneal diseases. They have only refractive errors and are otherwise healthy. Exclusion criteria for both groups include ocular allergies, contact lens wearing, history of eye surgery, and systemic or ocular diseases that might interfere tear film production or function [10]. Patients displayed ocular symptoms for at least 3 months and received no topical or systemic therapy for at least 4 weeks. Morning fasting values of testosterone, LH, FSH, DHEA-S, 17-OH-Prog, TSH, bound and unbound T3 and T4, prolactin, thyroglobulin, and cortisol were determined in 1 milliliter (mL) serum of patients and controls on the same day by using Vidas PC (bioMerieux, France) with macroELISA technique. Statistical evaluation was done by using SPSS 15.0 independent samples *t*-test.

## 3. Results

The mean score of ocular symptoms of patients with seborrheic MGD was 9.48 over 14.0, and the mean score of lid margin abnormalities was 2.98 over 3.0. SPK was not detected in both groups. The tear film BUT was in the normal range, and there were no loss of the meibomian glands in both groups. The patients had the score of meibomian gland expression either grade 0 or grade 1.  Table 1 discloses mean and standard deviation (SD) of serum levels of TSH, bound and unbound T3 and T4, FSH, LH, 17-OH-Prog, estrodiol, prolactin, testosterone, DHEA-S, cortisol, and thyroglobulin of the patients and controls in both genders. The mean (SD) of serum testosterone level was 2.27 (2.72) ng/mL in controls and 4.78 (3.67) ng/mL in patients (Table 1). The data mean (SD) of serum testosterone level in both genders of patients and controls was 3.52 (3.45) (Table 4). There was statistically significant difference of mean serum values of testosterone in both genders with respect to patients with seborrheic MGD and controls (*P* = 0.000, 95% confidence interval (CI) 1.22, 3.79; Table 4). The mean (SD) of serum DHEA-S level was 370.70 (146.50) *μ*g/mL in patients with seborrheic MGD and 238.50 (97.72) *μ*g/mL in controls (Table 1). The data mean (SD) of serum DHEA-S level in both genders of patients and controls was 304.6 (140.6) *μ*g/mL (Table 4). There was statistically significant difference of mean serum values of DHEA-S in both genders with respect to patients with seborrheic MGD and controls (*P* = 0.000, 95% CI 82.78, 181.63; Table 4).  Table 2 discloses the mean (SD) of serum hormone levels in female gender with respect to patients with seborrheic MGD and controls. The data mean of TSH in female gender of both patients and controls was 1.95 (1.17) *μ*IU/mL (Table 4). The mean value of TSH in female controls was 1.64 (0.89) *μ*IU/mL, and in female patients with seborrheic MGD it was 2.459 (1.40) *μ*IU/mL. There was statistically significant difference of mean serum values of TSH in female gender with respect to patients with seborrheic MGD and controls (*P* = 0.014, 95% CI 0.17, 1.48; Table 4). The data mean of serum prolactin level in female gender of both patients and control was 19.97 (9.74) ng/mL (Table 4). The mean value of prolactin in female controls was 17.79 (7.45) ng/mL and in female patients with seborrheic MGD was 23.51 (12.0) ng/mL (Table 2). There was statistically significant difference of mean serum values of prolactin in female gender with respect to patients with seborrheic MGD and controls (*P* = 0.043, 95% CI 0.19, 11.24; Table 4). The data mean of serum testosterone level in female gender of both patients and controls was 0.49 (0.29) ng/mL (Table 4). The mean value of testosterone in female controls was 0.37 (0.16) ng/mL and in female patients with seborrheic MGD was 0.69 (0.08) ng/mL (Table 2). There was statistically significant difference of mean serum values of testosterone in female gender with respect to patients with seborrheic MGD and controls (*P* = 0.001, 95% CI 0.15, 0.49; Table 4). The data mean of serum DHEA-S level in female gender of both patients and controls was 255.20 (122.70) *μ*g/mL. The mean value of DHEA-S in female controls was 212.07 (86.32) *μ*g/mL and in female patients with MGD was 325.57 (141.78) *μ*g/mL (Table 2). There was statistically significant difference of mean serum values of DHEA-S in female gender with respect to patients with seborrheic MGD and controls (*P* = 0.001, 95% CI 48.80, 178.19; Table 4).  Table 3 shows the mean (SD) of serum hormone levels in male gender with respect to the patients with seborrheic MGD and controls. The data mean of serum testosterone level in male gender of both patients and controls was 6.56 (2.28) ng/mL (Table 4). The mean value of serum testosterone in male controls was 5.376 (1.91) ng/mL and in patients with seborrheic MGD was 7.281 (2.20) ng/mL (Table 3). There was statistically significant difference of mean serum values of testosterone in male gender with respect to patients with seborrheic MGD and controls (*P* = 0.003, 95% CI 0.68, 3.14; Table 4). The data mean of serum DHEA-S level in male gender of both patients and controls was 354.0 (141.10) *μ*g/mL. The mean value of serum DHEA-S level in male patients with seborrheic MGD was 398.38 (144.6) *μ*g/mL, and in controls was 281.63 (102.0) *μ*g/mL. There was statistically significant differences of mean serum levels of DHEA-S in male gender with respect to patients with seborrheic MGD and controls (*P* = 0.003, 95% CI 40.42, 193.07; Table 4). No statistically significant differences of mean serum levels of the hormones including bound and unbound T3 and T4, FSH, LH, 17-OH-Prog, estrodiol, cortisol, and thyroglobulin in both genders were found between the patients with seborrheic MGD and controls (*P* > 0.05).

## 4. Discussion

This is the first time a case-control comparative study has been undertaken to assess the endocrine aspects of seborrheic MGD in a gender-based design. It represents the first report of increased serum levels of testosterone and DHEA-S in both genders with seborrheic MGD and increased serum levels of TSH and prolactin only in female gender with seborrheic MGD. Meibomian gland is considered as an androgen target organ [11]. Androgens (testosterone) are reported to control meibomian gland function, regulate the quality and/or quantity of lipids produced by this tissue, and promote the formation of the tear film's lipid layer [5, 11]. Previous studies related to androgen deficiency revealed significant and striking alterations in the lipid patterns of meibomian gland secretions, and it was considered to be an important etiologic factor in the pathogenesis of evaporative dry eye [4, 5]. However, testosterone excess is considered to provoke or aggravate seborrhea at a significance rate of *P* < 0.01 [12]. The effect of androgens on human skin is reported to increase sebaceous gland growth and differentiation, produce acne and seborrhea [13]. We demonstrated statistically significant increase of serum levels of testosterone in both genders with seborrheic MGD with respect to control genders. The mean score of ocular symptoms of the patients with seborrheic MGD was 9.48 over 14.0, and the mean score of lid margin abnormalities was 2.98 over 4.0. The value of serum testosterone was considered to be a significant factor affecting the severity of seborrehic MGD in both genders. Adrenal glands secrete large amounts of DHEA and DHEA-S which are then converted into potent androgens (testosterone and dehydrotestosterone) or estrogens by stereogenic enzymes in the peripheral sites that permit target tissues to adjust the formation and metabolism of androgens and estrogens to local requirements [14]. Increased serum levels of DHEA-S have been reported in patients with seborrheic dermatitis, acne vulgaris, alopecia, and hirsutism [15–17]. The aging process is paralleled by a dramatic decline in the serum concentrations of DHEA and DHEA-sulphate [14]. Serum levels of DHEA is reported to decrease from the age of 30 years [18]. In addition, there is a marked decline in the circulating levels of DHEA in postmenopausal women [18]. Our study includes the patients with seborrheic MGD at young age, mean age of 23, and age-matched controls. We demonstrate significant increase in serum levels of DHEA-S in both genders of patients with respect to control genders. We consider increase in serum values of DHEA-S in patients with seborrheic MGD could be used as diagnostic marker, and could be correlated with severity of the disease. Estrogens, glucocorticoids, and prolactin are also considered to influence sebaceous gland function by stimulating proliferation of sebocyte [19, 20]. Previous studies demonstrated that tear production in humans is correlated with prolactin [21]. The effect of prolactin on meibomian gland function has not been reported before. Our study revealed significant increase in serum levels of prolactin in female gender of patients with seborrheic MGD as compared to control gender. However, there was no significant increase in serum levels of prolactin in male patients with seborrheic MGD as compared to control gender. The correlation of serum prolactin level with seborrheic MGD remains to be determined. The function of TSH on sebocytes has not been reported before. Our study revealed significant increase serum levels of TSH only in female gender of the patients with seborrheic MGD with respect to the control gender. The correlation of serum levels of TSH with seborrheic MGD also remains to be determined in larger cohorts. No significant difference of serum levels of other hormones including bound and unbound T3 and T4, FSH, LH, progesteron, estrodiol, cortisol and thyroglobulin were found between patients with seborheic MGD and controls in both genders. In conclusion: Increased serum levels of testosterone and DHEA-S in both genders should be considered as diagnostic markers for seborrheic MGD and may affect severity of the disease. However, the correlations of serum levels of TSH and prolactin with seborrheic MGD need to be further investigated.

## Figures and Tables

**Table 1:** Descriptive statistics of serum hormone levels in both genders with respect to patients with seborrheic meibomian gland dysfunction and controls.

Hormones	Groups	*N*	Mean	SD
TSH (*μ*IU/mL)	Patient	50	1,9188	1,11642
	Control	50	1,7696	1,00285
Bound-T3 (nmol/L)	Patient	50	1,5930	,32533
	Control	50	1,7884	1,42732
Bound-T4 (nmol/L)	Patient	50	81,0106	9,74157
	Control	50	83,3822	10,77266
Unbound-T3 (pmol/L)	Patient	50	4,9398	,75224
	Control	50	4,8162	,80606
Unbound-T4 (pmol/L)	Patient	50	14,4654	2,17048
	Control	50	14,4144	2,42574
FSH (mIU/mL)	Patient	50	3,5184	2,00730
	Control	50	4,2894	1,95526
LH (mIU/mL)	Patient	50	4,1122	3,37226
	Control	50	3,8674	2,64136
17-OH-Prog (ng/mL)	Patient	50	2,2484	3,90230
	Control	50	3,8628	6,99213
Estrodiol (pg/mL)	Patient	50	69,2944	56,87763
	Control	50	79,4172	63,27935
Prolactin (ng/mL)	Patient	50	17,9796	9,88949
	Control	50	18,3808	8,63397
Testosterone (ng/mL)	Patient	**50**	**4,7764**	**3,66869**
	Control	**50**	**2,2714**	**2,71801**
Cortisol (ng/mL)	Patient	50	147,0022	55,22128
	Control	50	130,5280	53,70375
DHEA-S *μ*g/mL	Patient	**50**	**370,7088**	**146,50287**
	Control	**50**	**238,5034**	**97,72012**
Thyroglobulin (IU/mL)	Patient	50	11,9058	8,16335
	Control	50	11,0008	14,74698

DHEA-S: Dihydroepiandrosterone-sulphate, 17-OH-Prog: 17 Hydroxyprogesteron, FSH: Follicle stimulating hormone, LH: Lutenizing hormone, *N*: Number, TSH: thyroid stimulating hormone, T3: triiodothyronine, T4: thyroxine, SD: Standard deviation.

**Table 2:** Descriptive statistics of serum hormone levels in female gender with respect to patients with seborrheic meibomian gland dysfunction and controls.

Sex	Group		*N*	Mean		SD
			Statistic	Statistic	Std. Error	Statistic
F	Control	TSH (*μ*IU/mL)	**31**	**1,6361**	**,16040**	**,89308**
		Bound-T3 (nmol/L)	31	1,8371	,32359	1,80170
		Bound-T4 (nmol/L)	31	83,4255	1,77369	9,87551
		Unbound-T3 (pmol/L)	31	4,5261	,10682	,59474
		Unbound-T4 (pmol/L)	31	13,8426	,30131	1,67762
		FSH (mIU/ml)	31	4,5674	,34349	1,91249
		LH (mIU/ml)	31	4,3300	,57185	3,18394
		17-OH-Prog (ng/ml)	31	5,4384	1,52137	8,47062
		Estrodiol (pg/ml)	31	99,3897	12,72410	70,84477
		Prolactin (ng/ml)	**31**	**17,7935**	**1,33895**	**7,45495**
		Testosterone (ng/ml)	**31**	**,3687**	**,02907**	**,16186**
		Cortisol (ng/ml)	31	125,9610	9,50273	52,90897
		DHEA-S (*μ*g/ml)	**31**	**212,0700**	**15,50368**	**86,32082**
		Thyroglobulin (IU/ml)	31	12,6848	3,30031	18,37533
	Patients	TSH (*μ*IU/mL)	**19**	**2,4595**	**,32201**	**1,40359**
		Bound-T3 (nmol/L)	19	1,4242	,06133	,26734
		Bound-T4 (nmol/L)	19	78,5416	2,61172	11,38422
		Unbound-T3 (pmol/L)	19	4,4295	,13957	,60835
		Unbound-T4 (pmol/L)	19	13,5500	,54541	2,37738
		FSH (mIU/ml)	19	4,4558	,34066	1,48492
		LH (mIU/ml)	19	6,2158	1,04083	4,53688
		17-OH-Prog (ng/ml)	19	4,3626	1,33275	5,80933
		Estrodiol (pg/ml)	19	113,9679	16,43153	71,62339
		Prolactin (ng/ml)	**19**	**23,5121**	**2,75322**	**12,00099**
		Testosterone (ng/ml)	**19**	**,6895**	**,07690**	**,33520**
		Cortisol (ng/ml)	19	146,7295	13,17991	57,44987
		DHEA-S (*μ*g/ml)	**19**	**325,5653**	**32,52678**	**141,78094**
		Thyroglobulin (IU/ml)	19	13,6400	2,23048	9,72246

DHEA-S: dihydroepiandrosterone-sulphate, 17-OH-Prog: 17 hydroxyprogesteron, F: female, FSH: follicle stimulating hormone, LH: lutenizing hormone, *N*: number, Std: standard, SD: standard deviation, TSH: thyroid stimulating hormone, T3: triiodothyronine, T4: thyroxine,

**Table 3:** Descriptive statistics of serum hormone levels in male gender with respect to patients with seborrheic meibomian gland dysfunction and controls.

Sex	Group		*N*	Mean		SD
			Statistic	Statistic	Std. Error	Statistic
M	Control	TSH (*μ*IU/mL)	19	1,9874	,26434	1,15225
		Bound-T3 (nmol/L)	19	1,7089	,08107	,35338
		Bound- T4 (nmol/L)	19	83,3116	2,84108	12,38399
		Unbound-T3 (pmol/L)	19	5,2895	,20492	,89322
		Unbound-T4 (pmol/L)	19	15,3474	,71985	3,13776
		FSH (mIU/mL)	19	3,8358	,45658	1,99018
		LH (mIU/mL)	19	3,1126	,24352	1,06148
		17-OH-Prog (ng/mL)	19	1,2921	,34430	1,50077
		Estrodiol (pg/mL)	19	46,8305	6,18862	26,97555
		Prolactin (ng/mL)	19	19,3389	2,39230	10,42780
		Testosterone (ng/mL)	**19**	**5,3758**	**,43887**	**1,91299**
		Cortisol (ng/mL)	19	137,9795	12,75480	55,59687
		DHEA-S (*μ*g/mL)	**19**	**281,6316**	**23,40666**	**102,02727**
		Thyroglobulin (IU/mL)	19	8,2532	,92916	4,05011
	Patients	TSH (*μ*IU/mL)	31	1,5874	,13382	,74508
		Bound-T3 (nmol/L)	31	1,6965	,05705	,31762
		Bound-T4 (nmol/L)	31	82,5239	1,51352	8,42691
		Unbound-T3 (pmol/L)	31	5,2526	,11862	,66043
		Unbound-T4 (pmol/L)	31	15,0265	,33348	1,85673
		FSH (mIU/mL)	31	2,9439	,37505	2,08818
		LH (mIU/mL)	31	2,8229	,23447	1,30546
		17-OH-Prog (ng/mL)	31	,9526	,04296	,23916
		Estrodiol (pg/mL)	31	41,9139	2,32347	12,93656
		Prolactin (ng/mL)	31	14,5887	1,16484	6,48553
		Testosterone (ng/mL)	**31**	**7,2813**	**,39570**	**2,20316**
		Cortisol (ng/mL)	31	147,1694	9,83788	54,77501
		DHEA-S (*μ*g/mL)	**31**	**398,3774**	**25,97363**	**144,61507**
		Thyroglobulin (IU/mL)	31	10,8429	1,25798	7,00414

DHEA-S: dihydroepiandrosterone-sulphate, 17-OH-Prog: 17 hydroxyprogesteron, FSH: follicle stimulating hormone, LH: lutenizing hormone, M: male, N: number, TSH: thyroid stimulating hormone, T3: triiodothyronine, T4: thyroxine, Std: Standard, SD: Standard deviation.

**Table 4:** Descriptive statistics of serum hormone levels with respect to genders disclosing statistically significant differences of mean values between patients with seborrheic meibomian gland dysfunction and controls.

	Controls mean (SD)	Patients mean (SD)	*P* values (95% CI)	Controls and patients mean (SD)
All gender				
Testosterone (ng/ml)	2.27 (2.72)	4.78 (3.67)	*P* = 0.000 (1.22, 3.79)	3.52 (3.45)
DHEA-S (*μ*g/ml)	238.50 (97.70)	370.70 (146.50)	*P* = 0.000 (82.78, 181.63)	304.60 (140.60)
Female				
TSH (*μ*IU/mL)	1.64 (0.89)	2.46 (1.40)	*P* = 0.014 (0.17, 1.48)	1.95 (1.17)
Prolactin (ng/ml)	17.79 (7.45)	23.51 (12.00)	*P* = 0.043 (0.19, 11.24)	19.97 (9.74)
Testosterone (ng/ml)	0.37 (0.16)	0.69 (0.08)	*P* = 0.001 (0.15, 0.49)	0.49 (0.29)
DHEA-S (*μ*g/ml)	212.07 (86.32)	325.57 (141.78)	*P* = 0.001 (48.80, 178.19)	255.20 (122.70)
Male				
Testosterone (ng/ml)	5.38 (1.91)	7.28 (2.20)	*P* = 0.003 (0.68, 3.14)	6.56 (2.28)
DHEA-S (*μ*g/ml)	281.60 (102.0)	398.40 (144.60)	*P* = 0.003 (40.42, 193.07)	354.0 (141.10)

CI: confidence interval, DHEA-S: dihydroepiandrosterone-sulphate, TSH: thyroid stimulating hormone, SD: standard deviation.
